# Using Artificial Intelligence to Improve Pain Assessment and Pain Management: A Scoping Review

**DOI:** 10.1093/geroni/igab046.2409

**Published:** 2021-12-17

**Authors:** Meina Zhang, Linzee Zhu, Shih-Yin Lin, Keela Herr, Nai-Ching Chi

**Affiliations:** 1 University of Iowa, Iowa City, Iowa, United States; 2 New York University, New York, New York, United States; 3 University of Iowa, university of Iowa, Iowa, United States

## Abstract

Approximate 50 million U.S. adults experience chronic pain. It is a widely held view that pain has been linked to sleep disturbance, mental problems, and reduced quality of life. Uncontrolled pain has led to increased healthcare utilization, hospitalization, emergency visits, and financial burden. Recognizing, assessing, understanding, and treating pain can improve outcomes of patients and healthcare use. A comprehensive synthesis of the current use of AI-based interventions in pain management and pain assessment and their outcomes will guide the development of future clinical trials. This review aims to investigate the state of the science of AI-based interventions designed to improve pain management and pain assessment for adult patients. The electronic databases Web of Science, CINAHL, PsycINFO, Cochrane CENTRAL, Scopus, IEEE Xplore, and ACM Digital Library were searched. The search identified 2131 studies, and 18 studies met the inclusion criteria. The Critical Appraisals Skills Programme was used to assess the quality. This review provides evidence that machine learning, deep learning, data mining, and natural language processing were used to improve efficient pain recognition and pain assessment (44%), analyze self-reporting pain data (6%), predict pain (6%), and help physicians and patients to more effectively manage with chronic pain (44%). Findings from this review suggest that using AI-based interventions to improve pain recognition, pain prediction, and pain self-management is effective; however, most studies are pilot study which raises concerns about the generalizability of findings. Future research should focus on examining AI-based approaches on a larger cohort and over a longer period of time.

